# Light Ether Anæsthesia

**Published:** 1923-03

**Authors:** A. L. Flemming

**Affiliations:** Hon. Anæsthetist, Bristol Royal Infirmary


### LIGHT ETHER ANÆSTHESIA.

BY

A. L. Flemming, M.B., B.Ch.,

*Hon. Anæsthetist, Bristol Royal Infirmary.*

In ether anæsthesia the depth of narcosis employed is a far more important factor than appears to have been generally recognised.

The task of forming an accurate estimate of the respective virtues of deep and light ether anæsthesia is, however, by no means a simple problem ; the data obtainable from a comparison of operation results under the deep narcosis in vogue fifteen or twenty years ago with those of to-day do not afford much help, owing to the altered conditions obtaining in modern surgery. On the one hand, the " unfit " patient is nowadays often subjected to operations which formerly would have been regarded as of too severe a type for even the " fit " individual to stand ; on the other hand, improvements in surgical technique have done much in reducing the incidence of shock. In these circumstances it is naturally difficult to assess the proportion of credit for improvements which have occurred that is due to any one factor, such as a particular method of anæsthetisation.

My own experience has convinced me that with ether the light type of narcosis has many advantages over the deep type, and I believe that such doctrine is based upon sound principles. In fact, the mere probability that ether itself can produce symptoms of shock, by reason of its toxic action, suggests the need for careful limitation of dosage.

There are certain prejudices and factors which apparently prevent light ether anæsthesia from being used as generally as would otherwise be the case. Among these may be mentioned the following :—

1. A belief that deep anæsthesia affords better protection against shock.

2. A failure to recognise the potency of ether as a toxic agent.

3. The impression that deep anæsthesia is necessary for the production of relaxation suitable for abdominal work.

4. From the administrator's point of view there can be no doubt that the deep method is much the easier, especially in connection with abdominal surgery, in which class of case, however, it may be of the utmost importance to leave the patient, after operation, as free of the drug as possible.

Light anæsthesia may be defined as the stage in which the patient remains quiet, with the swallowing reflex in abeyance, the corneal reflex generally present, and in every other respect showing as little deviation from the normal as possible ; there should be no trace of cyanosis, no salivation, no suffusion of the skin, and only the slightest stimulation of respiration.

If we review these factors in detail, we find clinically every reason to discredit the belief that the protection against shock which is afforded by a general anæsthetic varies directly with the depth of narcosis.

Experience shows that it is the rule rather than the exception to see patients who have been lightly anæsthetised stand even the severest operations with a minimum of shock; and such result is quite in keeping with the experimental data upon which Crile[1] based his classical pronouncement "that provided anæsthesia is complete there is no advantage to be gained, from the point of view of shock, in deepening it."

Further evidence bearing on this problem is provided by the experiments conducted by Mann,[2] in which he failed to induce shock in anæsthetised animals by trauma to the limbs, even though severe and continued for as long as two hours.

In the same series of investigations it was shown that deep ether anæsthesia was itself capable of producing shock without the aid of trauma. Similar results were obtained by Rice Rich,[3] who in addition was able to prove that *light* ether anæsthesia for one hour protected against shock.

Mann,[4] working on ether tensions in the blood, found that a fall of blood pressure began to occur when ether saturation had reached that point at which the corneal reflex disappeared, a significant finding if we regard the disappearance of this reflex as an indication that the stage of light anæsthesia has been passed.

As to the toxicity of ether, and the consequent necessity for careful dosage, it is an easy matter to show experimentally that in the case of vegetable and animal organisms it acts as a definite protoplasmic poison, and we are indebted to Crile for demonstrating the degenerative changes it can produce in human tissues when exhibited in sufficiently large doses, or for long enough time. Clinically there is naturally difficulty in distinguishing between effects produced by the drug and those due to surgery.

When ether first took the place of chloroform in our practice, it brought with it a welcome sense of relief from the risk of what I may call "table" accidents, and we were wont to follow the old plan of keeping our patients with corneal and most of the other reflexes abolished. The resulting anæsthesia gave us a patient immobile during operation, but often very pale and nauseated and, in fact, suffering from many of the symptons of shock, after recovery to consciousness, even with the moderate surgery of those days. I believe that in this connection the surgeons actually robbed us of much of the blame to which we were entitled.

The growing popularity of ether brought with it an avalanche of new methods and machinery for its administration, and for each of these in turn was claimed the property of reducing or preventing shock. And the justice of the claim was soon established more or less definitely in each instance. But the one feature common to all these methods was a capacity for regulating the dose, and for maintaining an EVEN LIGHT ANÆSTHESIA.

The methods and apparatus referred to include: Roth Drager; Tyrrell; Stock; the Etherometer; Shipway; intravenous; intratracheal; combinations of ether with analgesics or with alkaloids, or with gas and oxygen.

These newer methods have enabled us to show that by dosimetric control of the strength of ether vapour administered we can practically eliminate that class of lung complication which is due directly to ether irritation, but we are still faced with chest sequelæ attributable indirectly to a state of lowered resistance to infection. The existence of such a factor is clearly demonstrated by experiences similar to those of Mikulicz, who owing to the frequency of chest complications abandoned ether for chloroform, with the result that the number of these sequelæ increased, and subsequently met with a still larger proportion of such cases when he substituted local anæsthesia for chloroform.

In the work of Hamburger,[5] Haines, and others we have proof that ether anæsthesia, in the early stages, stimulates and, in the later stages, diminishes phagocytosis and lowers the resistance to infection. Dr. Todd, at the Bristol Royal Infirmary, has kindly confirmed these statements for me by means of culture and by opsonic index. Experimentally it has been shown that etherised animals develop pneumonia when pneumococcus culture is introduced into their trachea, whereas normal animals resist the infection. All investigators in this field of research agree that the depressing effect is increased by prolonging the time of exposure to the vapour or by a deepening of the narcosis.

The question of muscular relaxation, especially in abdominal work, calls for a good deal of judgment on the part of the administrator. It is remarkable how often good relaxation can be obtained without loss of corneal reflex, provided not a trace of cyanosis be permitted. And not infrequently when more relaxation is called for the required effect is to be obtained more satisfactorily by a lightening rather than by a deepening of the narcosis, as Buxton[6] has pointed out.

That it should be important to preserve as much as is convenient of the muscle-tone seems only rational when we consider the part played by tone in the circulation of the blood and in the maintenance of blood pressure. Local anæsthesia, fortunately, is very helpful in this respect, and enables us to obtain relaxation of a single muscle, or group of muscles, without reducing the whole body to a state of atony, from which, in my experience, patients may be slow to recover.

The rather important question arises as to whether loss of muscle-tone may not be regarded as a cause as well as a result of "shock."

I have no doubt in my own mind that one reason which tends to make men chary about employing the lighter degrees of ether anæsthesia is the trouble it involves, such as the use of dosimetric apparatus, or, in the case of open ether, the need of counting the number of drops delivered per breath; nor is this all, for it is essential that the anæsthetist shall make himself intimately acquainted with the significance of even the minutest changes, both in the patient's reflexes and in the surgeon's facial expression.

I have seen a large number of patients with pulmonary tuberculosis undergo operation with the quiet anæsthesia of light ether without, as far as I know, a single case of activation; on the other hand, by administering gas and oxygen badly, in one instance at least, I undoubtedly stirred up a quiescent phthisis.

There may be no more general upset than is usual after gas, indeed I recall the case of an exsanguinated man, who after amputation through the upper third of the thigh for secondary hemorrhage, actually left the theatre smoking a cigarette.

### REFERENCES.

[1] *Proceedings of Seventeenth International Congress, London*, 1913.

[2] "Peripheral origin of Shock," *Johns Hopkins Hospital Bulletin*, 1914, xxv. 281.

[3] *Johns Hopkins Hospital Bulletin*, xxxviii. 79.

[4] "Vascular Reflexes with Ether Tensions," *Am Year Book*, 1917 p. 99.

[5] *Archives Neerlandaises*, 1911.

[6] *Clinical Journal*, 1921, p. 28

